# Asymmetric Dimethylarginine Disrupts Tumor Antigen Presentation in Breast Cancer

**DOI:** 10.3390/ijms26104482

**Published:** 2025-05-08

**Authors:** Mei Li, Yi-Ling Chen, Lilly M. Pearce, Amy M. Hammett, Falak H. Sharma, Derick S. Miller, Kuan-Hui E. Chen

**Affiliations:** 1 Department of Biological Sciences, Texas Tech University, Lubbock, TX 79409, USA; li38465@ttu.edu (M.L.); yiling100328@gmail.com (Y.-L.C.); lipearce@ttu.edu (L.M.P.); amhammet@ttu.edu (A.M.H.); fasharma@ttu.edu (F.H.S.); dermille@ttu.edu (D.S.M.); 2 Department of Electronic Engineering, National Kaohsiung University of Science and Technology, Kaohsiung 80778, Taiwan

**Keywords:** ADMA, antigen presentation, tumor phagocytosis, T cell activation, breast cancer

## Abstract

Asymmetric dimethylarginine (ADMA), an endogenous methylated amino acid, has been implicated in tumor progression; however, its influence on tumor immunity, particularly dendritic cell (DC) function and antigen presentation, remains unclear. In this study, we examined the effects of ADMA on tumor antigen uptake, processing, and presentation in DCs using the murine dendritic cell line DC2.4 as a model. Our results reveal that ADMA treatment significantly reduces the phagocytic uptake of tumor antigens derived from EO771 and Py230 breast cancer cell lysates. Additionally, ADMA exposure leads to a marked downregulation of key genes involved in antigen processing and presentation, including *MHC I*, *MHC II*, *TAP1*, *TAP2*, *ERp57*, and *CD80*. This suppression at the transcriptional level corresponds with decreased surface protein expression of MHC I, MHC II, and CD80, as confirmed by flow cytometry. Furthermore, ADMA-treated DC2.4 cells exhibit impaired tumor antigen presentation on their surface. Consequently, these functional impairments result in a diminished capacity to activate CD4^+^ T cells, as evidenced by a 41.18% decrease in CD25 expression and a 30.28% reduction in IFN-γ secretion. Similarly, CD8^+^ T cell activation is compromised, as indicated by a 32.26% decrease in IFN-γ production, although CD25 expression remains unaffected. Collectively, our findings identify ADMA as a potential immunosuppressive factor that disrupts antigen uptake, processing, and presentation in DCs, thereby modulating T cell activation. These insights suggest a potential mechanism through which ADMA may contribute to immune evasion within the tumor microenvironment.

## 1. Introduction

The nutritional landscape within the tumor microenvironment (TME) plays a crucial role in tumor progression. L-arginine, a semi-essential amino acid, has been shown to regulate cell growth [1,2]. Additionally, arginine metabolism influences macrophage polarization, determining whether they adopt an M1 phenotype, which promotes tumor elimination, or an M2 phenotype, which supports tumor growth [3]. Given these critical functions, numerous clinical trials have explored L-arginine deprivation as a potential cancer therapy [4,5,6,7,8,9]. However, the results have been inconsistent, suggesting the involvement of other metabolites in the TME that may impact the efficacy of arginine-based therapies.

Once taken up by cells, arginine can serve as a source for peptide synthesis or undergo various modifications. Since arginine is frequently found in the active sites of enzymes, its modification can influence numerous cellular processes [10]. Among these modifications, methylation and the resulting product, asymmetric dimethylarginine (ADMA), have been identified as significant [11]. ADMA, an endogenous inhibitor of nitric oxide synthase (NOS) [12], has gained increasing attention in tumors. Initially studied in the context of cardiovascular and metabolic diseases [13,14,15], ADMA is now recognized for its potential role in the TME. ADMA is a by-product of the arginine metabolic pathway, generated through the methylation of arginine residues in proteins by protein arginine methyltransferases (PRMTs) [16]. During proteolysis, these methylated arginine residues give rise to either asymmetric dimethylarginine (ADMA) or symmetric dimethylarginine (SDMA) [16].

Recent studies have highlighted the clinical significance of ADMA in cancer. Elevated serum ADMA levels have been observed in cancer patients, including those with breast cancer [17,18,19,20]. Our previous work demonstrated that breast tumor cells actively produce and release ADMA, potentially contributing to the increased circulating ADMA levels observed in patients [21]. Furthermore, a study by Hannemann et al., involving a cohort of 243 primary breast cancer patients, established a positive correlation between elevated serum ADMA levels, cancer recurrence, and mortality [20]. In addition to ADMA itself, research has also explored PRMT expression in tumor progression. Shi et al. showed that PRMT3 overexpression increased ADMA production and contributed to chemoresistance in hepatocellular carcinoma [22]. Consistently, Lei et al. demonstrated that PRMT3 knockdown reduced hepatic cancer cell growth and glycolysis [23]. Furthermore, Zou et al. found that a PRMT3 degrader effectively depleted ADMA levels, disrupted glycolysis, and induced cancer cell death in acute leukemia [24]. Collectively, these findings underscore the importance of the ADMA pathway in tumor development.

While correlations between ADMA levels, PRMT expression, and tumor progression have been established, the specific molecular mechanisms by which ADMA influences tumors and the TME remain largely unknown. The TME is composed of various stromal components, including immune cells that play a crucial role in both tumor progression and immune response. Macrophages have been reported to make up approximately 50% of tumor-infiltrating immune cells in both human and mouse studies [25,26], while T cells are among the most potent anti-tumor effectors [27,28]. However, the impact of ADMA on these immune populations remains largely unexplored. Our previous research demonstrated that tumor-derived ADMA can directly modulate macrophage polarization, driving the pro-tumorigenic M2 phenotype [21]. Additionally, dimethylguanidino valeric acid (DMGV), a metabolite of ADMA, has been shown to directly inhibit T cell proliferation by inducing mitochondrial reactive oxygen species (ROS) [29]. However, effective anti-tumor immunity relies not only on T cell expansion but also on cytokine production, which is driven by tumor antigen presentation by immune antigen-presenting cells. Antigen presentation is a complex, multi-step process that includes antigen recognition and uptake (phagocytosis), intracellular processing, selection and loading onto MHC molecules, and the transport of these complexes to the cell surface for presentation. Despite its potential immunomodulatory role, the effect of ADMA on antigen presentation remains unexamined. We therefore hypothesize that ADMA may suppress antigen presentation in dendritic cells, thereby impairing T cell activation.

In this study, we explore the role of ADMA in breast tumor immunology, focusing on its impact on tumor antigen presentation pathways. Specifically, we examine its effects on key stages, including tumor antigen phagocytosis, the expression of genes involved in antigen processing, surface presentation on MHC molecules, and subsequent T cell activation. Our findings reveal that ADMA exposure impairs both the phagocytic uptake of tumor antigens and the antigen-presenting capacity of immune antigen-presenting cells. While T cell clonal expansion remains unaffected, cytokine production by T cells is significantly reduced. These insights deepen our understanding of ADMA’s role in tumor immunity and underscore its potential as a biomarker and therapeutic target in breast cancer.

## 2. Results

### 2.1. ADMA Inhibits the Phagocytic Uptake of Tumor Antigens in Dendritic Cells

**Figure 1:**
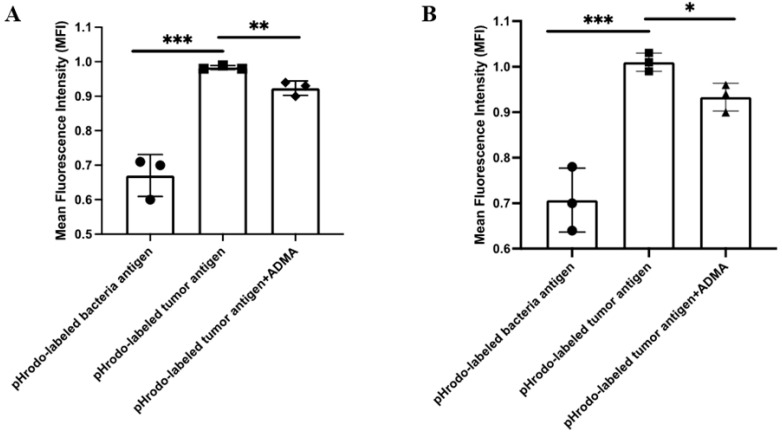
ADMA inhibits the phagocytic uptake of tumor antigens. The histograms illustrate the mean fluorescence intensity per dendritic cell analyzed by flow cytometry. Phagocytosis in DC2.4 cells was assessed using two types of pHrodo-labeled tumor antigens: EO771-derived tumor proteins (**A**) and Py230-derived tumor antigens (**B**), with bacterial antigens serving as a positive control. Data are pooled from at least three independent experiments and presented as mean ± SD. (*, *p* < 0.05; **, *p* < 0.01; ***, *p* < 0.001).

To assess the tumor antigen-presenting capacity, we first investigated whether ADMA affects the phagocytosis of tumor antigens by antigen-presenting cells. Dendritic cells are the most effective antigen-presenting cells; therefore, the mouse dendritic cell line DC2.4 was used as a model system to evaluate the impact of ADMA. Tumor antigens were prepared from two syngeneic mouse breast cancer cell lines, Py230 and EO771. To examine phagocytosis, tumor antigens from both cell lines, along with bacterial antigens as a control, were labeled with pHrodo dye, which fluoresces green in acidic environments. This property enables the detection of phagocytosis, as engulfed antigens are transported into the acidic environment of phagolysosomes. DC2.4 cells were exposed to tumor or bacterial antigens in the presence or absence of ADMA (300 ng/mL) for 24 h. Following incubation, cells were collected, and internal fluorescence—indicative of antigen uptake—was measured by flow cytometry to assess phagocytic activity. As shown in Figure 1, DC2.4 cells successfully phagocytosed both bacterial and tumor antigens derived from EO771 (Figure 1A) and Py230 (Figure 1B) cells. However, ADMA treatment led to a significant decrease in the phagocytic capacity of DC2.4 cells by 8% and 7.92% in response to EO771 and Py230 tumor antigens, respectively. To further confirm these results, we conducted real-time monitoring of tumor phagocytosis using the CellCyte X system, a fluorescence microscopy platform positioned inside the culture incubator that continuously captures cell images to track phagocytosis. In this experiment, tumor antigens were internalized into the phagolysosomes of DC2.4 cells, as quantified by pHrodo fluorescence. DC2.4 cells treated with ADMA exhibited lower fluorescence intensity, indicating a reduction in phagocytosis (Supplementary Figure S1).

### 2.2. Gene Expression Associated with Antigen Processing and Presentation in DC2.4 Cells Was Downregulated by ADMA

Once antigens are internalized, they undergo processing in order to be appropriately loaded onto MHC molecules for presentation. To determine whether ADMA affects intracellular antigen processing, we analyzed the expression of key genes involved in the antigen processing and presentation pathway using real-time PCR. These genes include TAP1 and TAP2, which transport cytoplasmic antigens degraded by proteasomes into the endoplasmic reticulum; MHC I and MHC II, which present processed antigens; ERP57, a protein disulfide isomerase that facilitates antigen editing; and CD80, a costimulatory molecule essential for effective T cell activation following antigen recognition.

**Figure 2:**
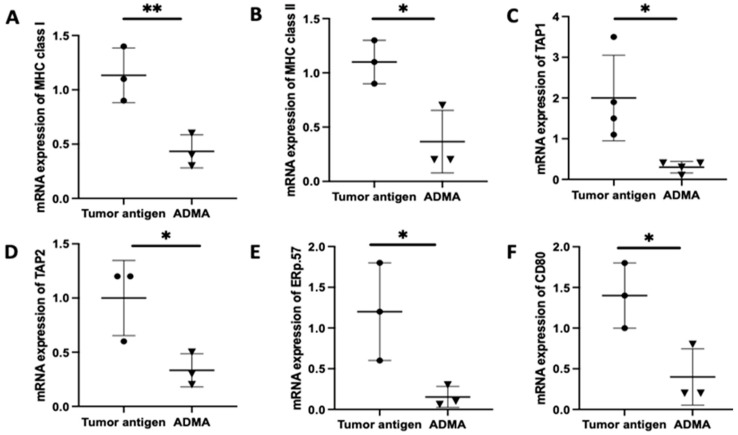
Gene expression associated with antigen processing and presentation in DC2.4 cells was downregulated by ADMA. DC2.4 cells were treated with tumor antigens derived from mouse breast cancer EO771 cells in the presence or absence of ADMA for six days, followed by RNA isolation and RT-qPCR analysis. The expression of six key genes involved in antigen processing and presentation, including MHC I (**A**), MHC II (**B**), TAP1 (**C**), TAP2 (**D**), ERp57 (**E**), and CD80 (**F**) was assessed. Data represent pooled results from at least three independent experiments and are presented as mean ± SD (*, *p* < 0.05; **, *p* < 0.01).

Antigen presentation by antigen-presenting cells to T cells is a complex process that typically unfolds over 5 to 7 days, involving antigen uptake, processing, and surface presentation on MHC molecules. To replicate this physiological process, we cultured DC2.4 cells with tumor antigens derived from EO771 or Py230 cells for six days in the presence or absence of ADMA. Following this incubation period, DC2.4 cells were harvested, and RNA was extracted for gene expression analysis. Comparative analysis revealed that ADMA treatment led to a significant downregulation of genes associated with antigen processing and presentation in DC2.4 cells exposed to EO771-derived tumor antigens (Figure 2). A similar pattern of gene suppression was also observed in DC2.4 cells exposed to Py230-derived tumor antigens in the presence of ADMA (Supplementary Figure S2). All six key genes examined—*MHC I*, *MHC II*, *TAP1*, *TAP2*, *Erp57*, and *CD80*—were significantly downregulated (*p* < 0.05) by ADMA in both models, highlighting ADMA’s regulatory influence on antigen presentation pathways.

**Figure 3:**
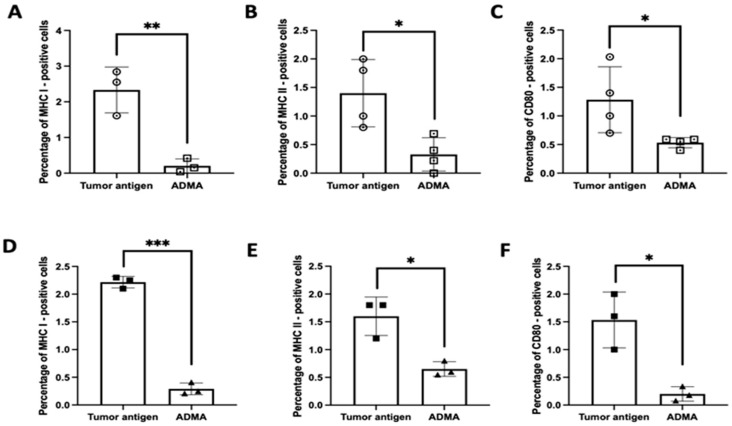
ADMA suppresses surface protein expression of MHC I, MHC II, and costimulatory molecule CD80 on DC2.4. Panels (**A**–**C**) display the protein expression levels of MHC I, MHC II, and CD80 in DC2.4 cells exposed to EO771-derived tumor antigens, while panels (**D**–**F**) show the corresponding marker expressions in DC2.4 cells exposed to Py230-derived tumor antigens, with or without ADMA treatment. Data represent pooled results from at least three independent experiments and are presented as mean ± SD (*, *p* < 0.05; **, *p* < 0.01; ***, *p* < 0.001).

To substantiate these RNA expression findings at the protein level, we assessed the surface expression of key antigen presentation markers—MHC I, MHC II, and CD80—using flow cytometry. Under identical experimental conditions, DC2.4 cells exposed to tumor antigens in the presence of ADMA exhibited a substantial reduction in the surface expression of these proteins compared to cells exposed to tumor antigens alone. This effect was consistent across both EO771-derived tumor antigens (Figure 3A–C) and Py230-derived tumor antigens (Figure 3D–F). These findings strongly suggest that ADMA disrupts antigen processing and presentation by downregulating both gene and protein expression of critical components involved in T cell activation. Given the pivotal role of antigen-presenting cells in shaping anti-tumor immune responses, our results highlight ADMA as a potential immunosuppressive factor that may contribute to tumor immune evasion.

### 2.3. ADMA Inhibits Tumor Antigen Presentation on the Surface of Dendritic Cells

So far, our findings indicate that dendritic cells exposed to ADMA exhibit impaired phagocytosis and antigen processing. Next, we aimed to determine whether these effects also lead to a reduction in tumor antigen presentation on the surface of dendritic cells. To facilitate the detection of surface antigen presentation, tumor antigens derived from EO771 and Py230 cells were chemically modified by conjugating a dibenzocyclooctyne (DBCO) group to their amine termini. Once internalized and processed, any presented tumor antigens on the cell surface could be detected using an azide-conjugated fluorescent probe, which specifically reacts with the DBCO moiety through click chemistry. Supplementary Figure S3 confirms that DBCO conjugation does not interfere with tumor antigen uptake.

**Figure 4:**
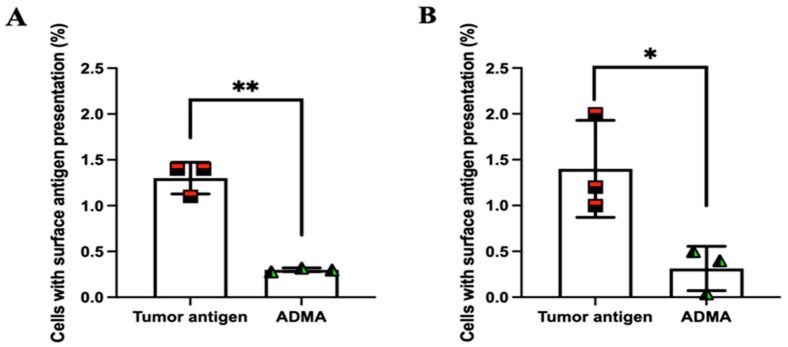
ADMA inhibits tumor antigen presentation on the surface of dendritic cells. DC2.4 cells were exposed to DBCO-labeled tumor antigens in the presence or absence of ADMA for six days, followed by flow cytometry analysis. The histograms compare the mean fluorescence intensity across groups based on flow cytometry data. To assess the impact of ADMA on antigen presentation, two types of DBCO-labeled tumor proteins were used: EO771-derived tumor proteins (**A**) and Py230-derived tumor proteins (**B**). Data represent pooled results from at least three independent experiments and are presented as mean ± SD (*, *p* < 0.05; **, *p* < 0.01).

Following the established protocol, DC2.4 cells were exposed to DBCO-labeled tumor antigens in the presence or absence of ADMA for six days. After incubation, cells were harvested and incubated with an azide-conjugated fluorescent probe. Unbound azide molecules were removed by washing with DPBS, and surface fluorescence was analyzed by flow cytometry. As shown in Figure 4, DC2.4 cells exhibited significantly reduced surface presentation of tumor antigens by 76.92% and 77.62% from EO771 (Figure 4A) and Py230 (Figure 4B), respectively, after six days of ADMA treatment. These results are consistent with our previous observations, further supporting the notion that ADMA disrupts tumor antigen uptake and processing, thereby impairing antigen presentation on the cell surface.

### 2.4. ADMA Impacted T Cell Activation in Different Ways

Since ADMA impairs the tumor antigen presentation capacity of dendritic cells, we next investigated whether this reduction in antigen presentation affects subsequent T cell activation. To address this, we conducted an ex vivo T cell activation assay. In this experiment, tumor antigens (derived from EO771 or Py230), DC2.4 cells, and splenic T cells were all syngeneic from the C57Bl/6 background. Following the established protocol, DC2.4 cells were exposed to tumor antigens from either EO771 or Py230 in the presence or absence of ADMA for six days. On day 7, these DC2.4 cells were harvested and co-cultured with splenic T cells for an additional 7 days.

**Figure 5:**
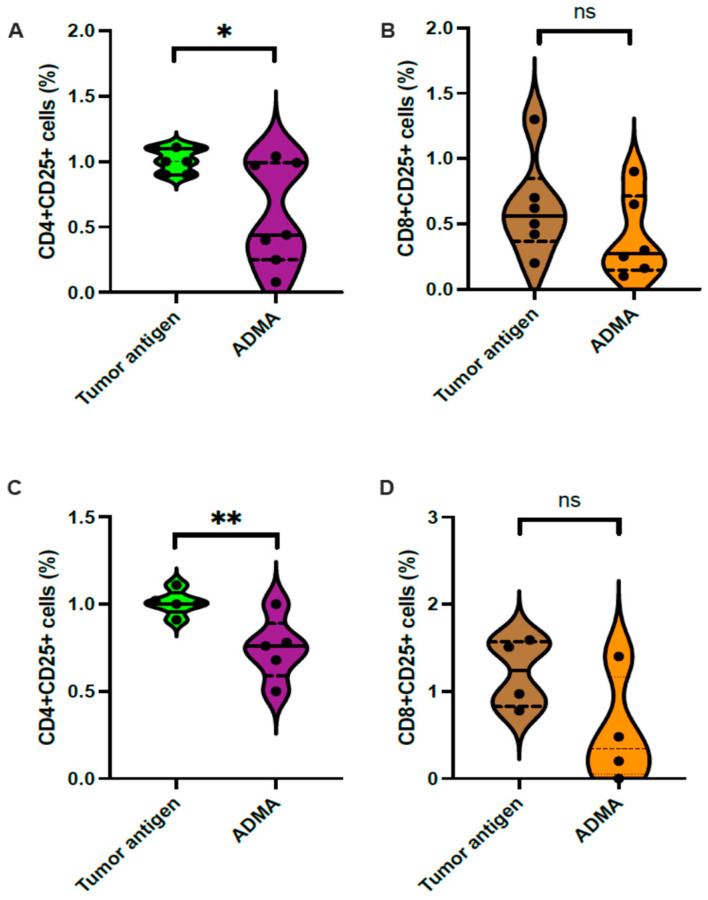
CD25 expression was selectively altered in splenic CD4^+^ T cells following antigen presentation by ADMA-treated DC2.4 cells. The percentage of CD25-expressing T cells was assessed by flow cytometry. A reduced percentage of CD4^+^ T cells was observed following antigen presentation by ADMA-treated DC2.4 cells presenting antigens from EO771 (**A**) and Py230 (**C**). In contrast, no significant difference was detected in the percentage of CD25-expressing CD8^+^ T cells after antigen presentation by ADMA-treated DC2.4 cells presenting antigens from EO771 (**B**) and Py230 (**D**). Data were pooled from at least three independent experiments and are presented as mean ± SD (*, *p* < 0.05; **, *p* < 0.01; ns, no statistical significance).

Activated T cells are known to upregulate the Interleukin-2 (IL2) receptor alpha subunit (CD25), making CD25 expression a reliable marker for T cell activation, which was assessed via flow cytometry. The results showed a significant reduction in the CD4^+^CD25^+^ population (Figure 5A) but no change in the CD8^+^CD25^+^ population (Figure 5B) among splenic T cells co-cultured with DC2.4 cells previously exposed to EO771 tumor antigens in the presence of ADMA. Similarly, a reduction in CD4^+^CD25^+^ T cells was observed when DC2.4 cells were exposed to Py230 tumor antigens with ADMA treatment (Figure 5C), while the CD8^+^CD25^+^ T cell population remained unchanged (Figure 5D).

**Figure 6:**
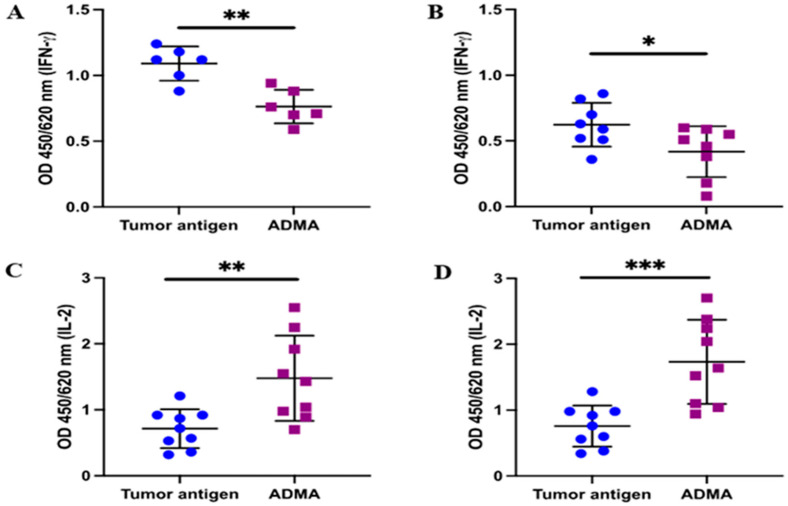
Antigen presentation by ADMA-treated DC2.4 cells modulates cytokine production in T cells. DC2.4 cells were exposed to tumor antigens derived from EO771 (**A**,**C**) and Py230 (**B**,**D**) cell lines for six days, with or without ADMA treatment. Supernatants were collected to assess cytokine secretion by primed T cells using ELISA, specifically measuring IFN-γ (**A**,**B**) and IL-2 (**C**,**D**). Data were pooled from at least three independent experiments and are presented as mean ± SD (*, *p* < 0.05; **, *p* < 0.01; ***, *p* < 0.001).

In addition to the upregulation of CD25 in activated T cells, the production of key cytokines such as interleukin-2 (IL-2) and interferon-gamma (IFNγ) plays a crucial role in maintaining the effector function of activated T cells. To further validate the T cell activation results based on CD25 expression, we assessed the secretion of IL-2 and IFNγ. Following the established protocol, conditioned medium was collected after the 7 day co-culture of splenic T cells with DC2.4 cells that had been pre-exposed to tumor antigens in the presence or absence of ADMA. The levels of IL-2 and IFNγ in the conditioned medium were then measured using ELISA. As expected, IFNγ production was reduced in the conditioned medium from co-cultures where DC2.4 cells had been exposed to EO771-derived antigens (Figure 6A) or Py230-derived antigens (Figure 6B) in the presence of ADMA. Interestingly, IL-2 levels increased significantly by 2.07-fold and 2.29-fold when ADMA-treated DC2.4 cells were exposed to EO771 or Py230 tumor antigens, respectively.

As IFNγ and IL2 are essential for effector function and clonal expansion, respectively, of activated T cells, these findings indicate that ADMA impairs T cell activation through reducing IFNγ production, likely due to its upstream inhibitory effect on dendritic cell antigen presentation.

### 2.5. ADMA Stimulated T Cells but Not Dendritic Cell Proliferation

Our primary objective was to determine whether ADMA affects dendritic cell function. Therefore, dendritic cells were exposed to ADMA only during the stages of antigen acquisition, processing, and surface presentation. Following this period, co-culture experiments with T cells were conducted in the absence of ADMA to specifically assess the effects of impaired antigen presentation on subsequent T cell activation, without direct ADMA influence on T cells.

Thus far, we have demonstrated that ADMA disrupts dendritic cell function at multiple stages, including the early phase of antigen phagocytosis, the intermediate stage of antigen processing and editing, and the later stage of surface antigen presentation. Given these functional impairments, we also explored whether ADMA might influence dendritic cell proliferation, potentially exacerbating its impact on dendritic cell viability. To investigate this, we continuously monitored dendritic cell growth in real time using the CellCyte X imaging system, which performed automated cell counting every two hours within the culture incubator over a six-day period. Our analysis showed no significant differences in cell proliferation between control and ADMA-treated dendritic cells (Supplementary Figure S4). These findings indicate that while ADMA alters dendritic cell function, it does not affect their growth or survival.

Furthermore, T cells within the local tumor microenvironment (TME) may also be exposed to ADMA. Since T cell activation relies on proper antigen presentation by antigen-presenting cells, ADMA alone would not directly trigger T cell activation. Therefore, we focused on the potential effects of ADMA on T cell proliferation. To assess this, we used the human Jurkat T cell line as a model and employed two complementary methods to measure proliferation.

**Figure 7:**
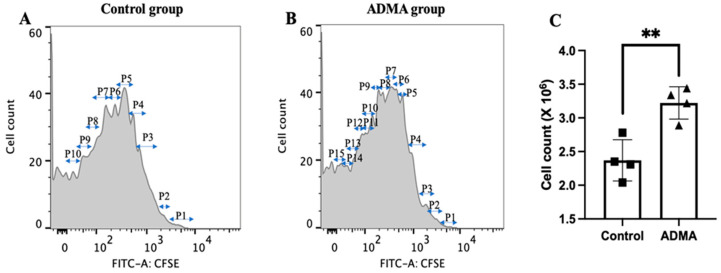
ADMA enhances T cell proliferation. T cell division was evaluated using CFSE staining, with flow cytometry analysis revealing a higher number of T cell divisions in the ADMA-treated group (**B**) compared to the negative control group (**A**). Consistently, direct cell counting confirmed a significant increase in T cell numbers following ADMA treatment compared to the negative control (**C**). Data are pooled from at least three independent experiments and presented as mean ± SD (** *p* < 0.01).

First, we used CFSE, a fluorescent dye that persists for 7–10 days. As T cells divide, fluorescence intensity decreases by half in each daughter cell, allowing cell division tracking via flow cytometry. As shown in Figure 7, ADMA treatment increased T cell proliferation, as evidenced by a greater number of fluorescence-halving events in the ADMA-treated group (Figure 7B) compared to the control group (Figure 7A). To further validate this result, we conducted a direct cell count, which confirmed that ADMA stimulation led to a significant increase in T cell numbers compared to the control group (Figure 7C).

## 3. Discussion

With a growing body of research linking elevated ADMA levels to cancer patients, ADMA has garnered increasing attention for its potential involvement in tumor progression. However, its impact on tumor stromal cells, particularly immune cell populations, remains underexplored. In this study, we investigated the effects of ADMA on tumor immune responses, with a specific focus on antigen-presenting dendritic cells (DCs), which play a critical role in initiating T cell-mediated anti-tumor immunity. Our findings demonstrate that ADMA disrupts multiple stages of dendritic cell function, including: (a) impaired phagocytosis of tumor antigens, (b) reduced cross-presentation of antigens to T cells, (c) diminished antigen processing and editing, and (d) decreased surface expression of antigen-presenting molecules. As a consequence of these alterations in dendritic cell function, T cell activation was significantly impaired, primarily due to a reduction in IFN-γ production. These findings suggest that ADMA may serve as an immunosuppressive factor within the tumor microenvironment, contributing to immune evasion by limiting effective antigen presentation and subsequent T cell activation.

Our findings suggest that ADMA-treated dendritic cells differentially modulate the activation of CD4^+^ and CD8^+^ T cells. In CD4^+^ T cells, antigen presentation by ADMA-exposed dendritic cells led to reduced CD25 expression, decreased IFN-γ production, and increased IL-2 secretion. CD25, also known as IL-2 receptor alpha (IL2RA), is a key component of the high-affinity IL-2 receptor (IL-2R), which consists of IL2RA (CD25), IL2RB, and IL2RG [30]. In contrast, IL-2R composed only of IL2RB and IL2RG forms a lower-affinity receptor for IL-2 [30]. The reduced expression of CD25 in CD4^+^ T cells may limit their differentiation and proliferation by restricting access to IL-2. Previous studies have shown that mice deficient in CD25 exhibit elevated serum IL-2 levels, likely due to an impaired ability to bind and utilize IL-2 efficiently through the high-affinity receptor [30]. Similarly, in our study, the increased IL-2 levels observed in the conditioned medium may reflect a compensatory response to the shift toward a low-affinity IL-2 receptor. Moreover, IL2 is essential to drive FoxP3 expression [31] and inducible T-regulatory precursors are characterized by a CD4^+^CD25^−^FoxP3^+^ phenotype [32,33]. This raises the possibility that ADMA-treated dendritic cells may also facilitate the induction of tumor-associated Tregs through a dendritic cell–mediated mechanism. Further studies are warranted to explore the role of ADMA in the generation of inducible Tregs, particularly within the tumor microenvironment. IFN-γ plays a crucial role in regulating CD4^+^ T cell effector functions [34,35]. Thus, the interaction between naïve T cells and ADMA-exposed dendritic cells likely results in impaired T cell differentiation, reduced IL-2-dependent expansion, and diminished effector activity. These findings highlight the potential immunosuppressive role of ADMA in modulating T cell responses within the tumor microenvironment.

In contrast, CD8^+^ T cells did not exhibit reduced CD25 expression following antigen presentation by ADMA-treated dendritic cells. However, similar to CD4^+^ T cells, CD8^+^ T cells also showed increased IL-2 production and decreased IFNγ secretion. These differences in T cell responses may be attributed to variations in the extent of antigen presentation via MHC I and MHC II pathways. Extracellular antigens acquired through phagocytosis are transported to an endosomal compartment known as the phagolysosome where MHC II is predominantly present [36]. Within this acidic environment, enzymatic degradation processes break down the antigens, which are then loaded onto MHC II molecules and transported to the cell surface for presentation. Given that MHC II primarily interacts with CD4^+^ T cells, any disruption in this pathway is expected to have a greater impact on CD4^+^ T cell activation compared to CD8^+^ T cells. Consistent with this expectation, our results demonstrate that ADMA-treated DC2.4 cells predominantly affected CD4^+^ T cell activation, further supporting the notion that ADMA impairs antigen processing and presentation through the MHC II pathway.

However, it is also reported that a small proportion of antigens in the phagolysosomes can be leaked out to cytoplasm where they were degraded in proteasomes, transported to endoplasmic reticulum via TAP1/2, edited by ERp57, and then loaded onto MHC I which eventually presented on the cell surface [37]. This process is known as cross priming or cross presentation. Not many antigen-presenting cells are able to perform cross priming. DC2.4 cells have been shown to perform cross presentation well [38,39,40]. Consistent with this, we showed that ADMA impacted both MHC I and II and many genes involved in cross-priming related processing and the surface antigen presentation on both MHC I and II even when the tumor antigens are extracellular. This also explains why CD8^+^ T cells are also partially affected by ADMA-stimulated dendritic cells.

Our findings demonstrate that ADMA not only impairs tumor antigen presentation on both MHC I and MHC II but also downregulates ERp57 expression. ERp57 is a crucial thiol oxidoreductase involved in antigen editing, a process that enhances antigen presentation stability [41]. During an immune response, peptides degraded by the proteasome are transported into the endoplasmic reticulum, where they are loaded onto MHC I molecules. ERp57 plays a key role in this process by facilitating the removal of low-affinity peptides and ensuring that only high-affinity antigens remain bound to MHC I, thereby prolonging antigen presentation. The duration of antigen presentation directly influences the strength of T cell activation [42]. A more stable and prolonged antigen-MHC I interaction enhances T cell activation, leading to a more robust immune response. Although ERp57 plays a crucial role in antigen editing—contributing to the proper folding and stability of peptide–MHC class I complexes, and thereby supporting sustained antigen presentation—its involvement is often overlooked and is rarely evaluated or reported in the majority of immunological studies, potentially leaving a gap in our understanding of the mechanisms underlying effective immune responses. Our results suggest that the reduced T cell activation observed when co-cultured with ADMA-treated dendritic cells is likely due not only to a decreased quantity of antigen presented by dendritic cells but also to the instability of antigen presentation caused by diminished ERp57 expression and impaired antigen editing.

Our findings also suggest that ADMA influences T cell proliferation. As a methylated form of arginine, ADMA utilizes multiple transporters to enter cells, including the same transporter (CAT2) as L-arginine [43]. Notably, ADMA can be metabolized into citrulline, a metabolite known to promote T cell proliferation [44]. Therefore, ADMA may serve as an alternative source of citrulline, particularly under conditions of arginine limitation. While ADMA may contribute to T cell proliferation, it could negatively affect T cell activation via its impact on TCR complex formation. L-arginine is essential for the expression of the CD3 zeta chain in T cells, which plays a critical role in regulating T cell activation and immune responses [45]. Since ADMA competes with L-arginine for cellular uptake [46,47], it may reduce intracellular arginine availability, potentially resulting in incomplete TCR assembly and impaired T cell activation. Moreover, several aspects of ADMA’s impact on T cells remain unclear, including its effect on the induction of arginine transporters, how transporter expression correlates with T cell activation status, and whether ADMA influences arginine transporter expression on other stromal cells, such as myeloid-derived suppressor cells [48]. Further research is necessary to investigate these possibilities.

## 4. Materials and Methods

### 4.1. Cell Culture and Treatment

Mouse dendritic cells (DC2.4; SCC142, Sigma-Aldrich, St. Louis, MO, USA), mouse triple-negative breast cancer cells Py230 (CRL-3297™, ATCC, Manassas, VA, USA), and mouse breast cancer cells EO771 (CRL-3461™, ATCC), which have been variably classified as either luminal B [49] or triple-negative breast cancer [50] in the literature, were cultured in complete RPMI 1640 medium (Cat. #10-040-CV, Corning, Corning, NY, USA) supplemented with 10% fetal bovine serum (FBS; 10082-147, ThermoFisher Scientific, Waltham, MA, USA) and 1% penicillin–streptomycin (SV30010, Cytiva, Marlborough, MA, USA). Jurkat T cells (TIB-152, ATCC) were maintained under the same conditions and used to assess the direct effects of ADMA on T cell proliferation. Mouse splenocytes, kindly provided by Lauren E. (see Acknowledgements), were also cultured in the same medium and served as an ex vivo model for T cell activation. All cells were maintained at 37 °C in a humidified incubator with 5% CO_2_.

### 4.2. Tumor Antigen Preparations

Two types of tumor antigens were prepared for the study. EO771 and Py230 cells were cultured to approximately 85–90% confluency, after which cell lysates were collected in sterile Dulbecco’s phosphate-buffered saline (DPBS) using repeated freeze-thaw cycles for protein extraction, as previously described [51]. Following protein isolation and quantification, the tumor proteins were conjugated with a DBCO crosslinker (C20039, ThermoFisher Scientific) at a ratio of 0.1 mg of protein to 4 × 10^4^ µM of DBCO. Excess, unbound DBCO was removed using filtration columns (PIA44297, ThermoFisher Scientific). These conjugated proteins are referred to in this study as DBCO-labeled tumor antigens. For phagocytosis assays, tumor antigens were also labeled with pHrodo™ Green STP ester (Cat. #P35369, Invitrogen, Carlsbad, CA, USA), a pH-sensitive fluorescent dye that emits green fluorescence in acidic environments such as phagolysosomes. Conjugation was performed by incubating 1.5 mg of tumor protein with 0.027 mg of pHrodo™ Green STP ester (P35369, ThermoFisher) at room temperature for 1 h.

### 4.3. Measurement of Phagocytosis

To evaluate the impact of ADMA on the phagocytic uptake of tumor antigens, DC2.4 cells were seeded in six-well plates at a density of 1 × 10^6^ cells per well and treated with either pHrodo™ Green Zymosan A BioParticles™ conjugate (bacterial antigen as control; Cat. #P35365, Invitrogen) at 0.018 mg/mL or pHrodo™ Green STP ester-labeled tumor antigens derived from EO771 and Py230 cells at 0.1 mg/mL. Treatments were conducted for 24 h in the presence or absence of ADMA. DC2.4 cells exposed to the pHrodo™ Green Zymosan A BioParticles™ alone served as a positive control to validate the functionality of the phagocytosis assay system. Phagocytosis of the labeled zymosan and tumor antigens was quantified using Cellcyte X (CYTENA, Boston, MA, USA) for imaging analysis and flow cytometry (BSN-ZS-AS7S, Conduct Science, Skokie, IL, USA) for quantitative assessment. The CellCyte X system has a resolution of 0.345 µm/pixel at 10× objective magnification. Images were captured every 5 min over a 24 h period to monitor phagocytosis.

### 4.4. RNA Isolation and Q-RT PCR Analysis

To investigate the expression of genes involved in antigen processing and presentation, DC2.4 cells were treated with DBCO-labeled tumor antigens in the presence or absence of ADMA for 6 days. Total RNA was isolated from the cultured cells using an RNA isolation kit (Cat. #2775172, ThermoFisher Scientific) following the manufacturer’s instructions. Complementary DNA (cDNA) was synthesized using the Synthesis cDNA Kit (Cat. #170-8891, Bio-RAD, Hercules, CA, USA). Quantitative real-time PCR (qRT-PCR) was then performed using SYBR Green qPCR Master Mix (Cat. #73112-0500, VWR, Radnor, PA, USA) on a OpenPCR system (A1005, Chai Bio, Santa Clara, CA, USA) for gene expression analysis. The PCR conditions were as follows: initial denaturation at 95 °C for 3 min, followed by 40 cycles of 95 °C for 30 s and 60 °C for 30 s. Primer sequences used in this study are listed in Supplementary Table S1.

### 4.5. Flow Cytometry

Flow cytometry was utilized across multiple experimental assays, including phagocytosis analysis, antigen presentation assessment, and T cell activation studies.

For phagocytosis analysis, 5 × 10^6^ DC2.4 cells were incubated for 24 h with either pHrodo™ Green zymosan A BioParticles™ conjugate or pHrodo-labeled tumor antigens (EO771 and PY230), in the presence or absence of ADMA. The pHrodo-labeled antigens emit green fluorescence upon internalization into the acidic environment of phagolysosomes. The mean fluorescence intensity (MFI) per dendritic cell was measured and used as an indicator of phagocytic activity.

For antigen presentation studies, DC2.4 cells were treated with DBCO-labeled tumor antigens (EO771 and PY230), with or without ADMA, for 6 days. The cells were then incubated with 0.2 μg of biotin-azide (Cat. #3020, AAT Bioquest, Pleasanton, CA, USA) at 4 °C for 30 min to label surface-presented antigens through azide–DBCO interaction. After washing, the cells were stained with fluorophore-conjugated antibodies against biotin (Cat. #53-9895-82, ThermoFisher Scientific), MHC I (Cat. #12-5958-82, Invitrogen), MHC II (Cat. #25-5321-82, Invitrogen), and CD80 (Cat. #MA5-28657, Invitrogen), each at 0.2 μg per sample for 30 min at 4 °C. The percentage of cells presenting antigens or expressing MHC I, MHC II, and CD80 was analyzed by flow cytometry.

For T cell activation, ADMA-pretreated DC2.4 cells were co-cultured with mouse-derived splenocytes for an additional 7 days. The cells were then harvested and resuspended in 100 μL of 1% BSA. To reduce non-specific antibody binding through the Fc receptor, 1 μg of Fc block (Cat. #564220, BD Pharmingen, San Diego, CA, USA) was added and incubated for 5 min at 4 °C. After washing with 1% BSA, the cells were resuspended in 200 μL of 1% BSA for downstream staining with fluorescently labeled antibodies targeting CD25 (Cat. #60-0251-U100, TONBO Biosciences, San Diego, CA, USA), CD4 (Cat. #1540-10, SouthernBiotech, Birmingham, AL, USA), and CD8 (Cat. #55-0081-U100, TONBO Biosciences), each at 0.2 μg per antibody for 30 min at 4 °C. After staining, the cells were washed, centrifuged, and resuspended in 1% BSA for analysis using a flow cytometer (BSN-ZS-AS7S, Conduct Science). Samples treated without DBCO-labeled tumor antigens and ADMA served as negative controls.

### 4.6. Enzyme-Linked Immunosorbent Assay (ELISA)

Cytokine levels secreted by activated mouse-derived splenocytes were quantified using IFN-γ (Cat. #430816, BioLegend, San Diego, CA, USA) and IL-2 (Cat. #431001, BioLegend) ELISA kits, following the manufacturer’s protocols. DC2.4 cells were first treated with DBCO-labeled tumor antigens, with or without ADMA, for 6 days, and subsequently co-cultured with mouse-derived splenocytes for an additional 7 days. Following co-culture, supernatants were collected and analyzed via ELISA. Absorbance readings at 450 nm and 620 nm were measured using a microplate reader (423555, Biolegend). All experiments were conducted in triplicate.

### 4.7. Cell Proliferation Measurement

The impact of ADMA on dendritic cell and T cell growth was evaluated using direct cell counting and Carboxyfluorescein succinimidyl ester (CFSE, Cat. #C34554, ThermoFisher) staining. CFSE is a fluorescent dye that permeates cells and covalently binds to intracellular components. As cells divide, the fluorescence intensity is equally distributed between daughter cells, allowing for the assessment of cell division by tracking the progressive halving of fluorescence. A total of 5 × 10^6^ DC2.4 or Jurkat T cells were stained with CFSE, washed, and then cultured in fresh plates with or without 300 ng/mL of ADMA for 7 days. Flow cytometry was used to analyze the halving of fluorescence intensity, which was indicative of the number of cell divisions. For direct cell counting, the Cellcyte X system (ECCX0200, Echo, San Diego, CA, USA) was employed. This live-cell imaging platform operates within the incubator, capturing images and monitoring cell proliferation automatically at user-defined time points. Additionally, 5 × 10^3^ DC2.4 or Jurkat T cells were cultured in a 96-well plate with 300 ng/mL of ADMA for 7 days, and cell numbers were monitored every 2 h using the Cellcyte X system until the experiment was completed.

### 4.8. Statistical Analysis

All experiments were repeated at least three times, and data are presented as mean ± SD, with significance considered at *p* ≤ 0.05. Statistical analysis was primarily performed using a paired *t*-test for comparisons between two groups. For comparisons with more than two groups, one-way ANOVA followed by post-hoc Tukey HSD tests was used. Two-tailed tests were applied for all data analyses. Graphs were generated using GraphPad Prism software (version 8.01).

## 5. Conclusions

In conclusion, our study demonstrates that ADMA plays a significant role in modulating the tumor microenvironment by suppressing the function of antigen-presenting cells, particularly dendritic cells. ADMA treatment impairs key processes involved in antigen uptake, processing, and presentation, leading to a reduced capacity to activate T cells. This suppression of antigen presentation, along with the impact on T cell activation, suggests that ADMA may contribute to immune evasion in tumors, providing a potential target for therapeutic strategies aimed at enhancing anti-tumor immunity.

## Data Availability

The dataset supporting the conclusions of this article is available from the corresponding author.

## Supplementary Materials

The following supporting information can be downloaded at: https://www.mdpi.com/article/10.3390/ijms26104482/s1.

## Abbreviations

The following abbreviations are used in this manuscript:

ADMA	asymmetric dimethylarginine
DC	dendritic cells
TME	tumor microenvironment
NOS	nitric oxide synthase
PRMT	protein arginine methyltransferase
ROS	reactive oxygen species
DMGV	dimethylguanidino valeric acid
DBCO	dibenzocyclooctyne
IFNγ	interferon-gamma
IL2	interleukin-2
IL2R	interleukin-2 receptor
DPBS	Dulbecco’s phosphate-buffered saline
ELISA	Enzyme-linked Immunosorbent Assay
CFSE	Carboxyfluorescein succinimidyl ester
