# Supplementary material for: Asymmetric Dimethylarginine Disrupts Tumor Antigen Presentation in Breast Cancer
Source: Int J Mol Sci. 2025 May 8;26(10):4482. doi: 10.3390/ijms26104482 (PMC12111280; doi:10.3390/ijms26104482)
Supplement: Supplementary file 1 [file ijms-26-04482-s001.zip › ijms-3599744-supplementary.pdf]

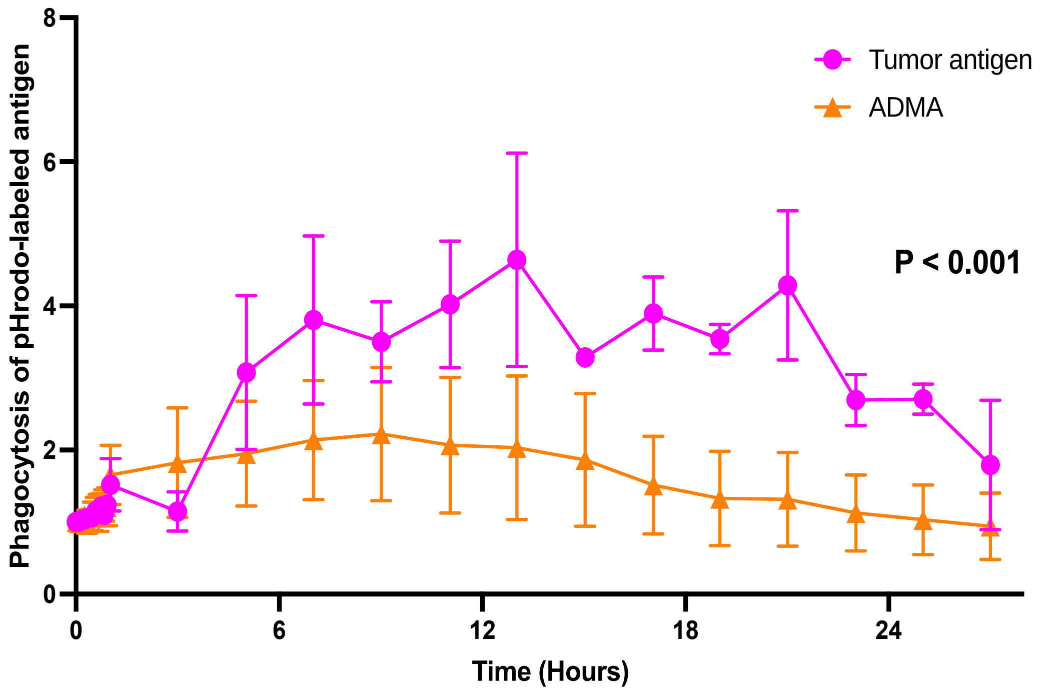

Supplementary Figure S1. Real-time Monitoring of Reduced Tumor Antigen Phagocytosis by ADMA Treatment in DC2.4 Cells. DC2.4 cells were seeded in a 6-well plate (1×10^6 cells/well) for 24 hours before being treated with pHrodo-labeled tumor antigens, with or without ADMA, for an additional 24 hours. The fluorescence signal from pHrodo™ Green STP ester-labeled tumor antigens that were taken into phagolysosomes was continuously monitored using the CellCyte X system.

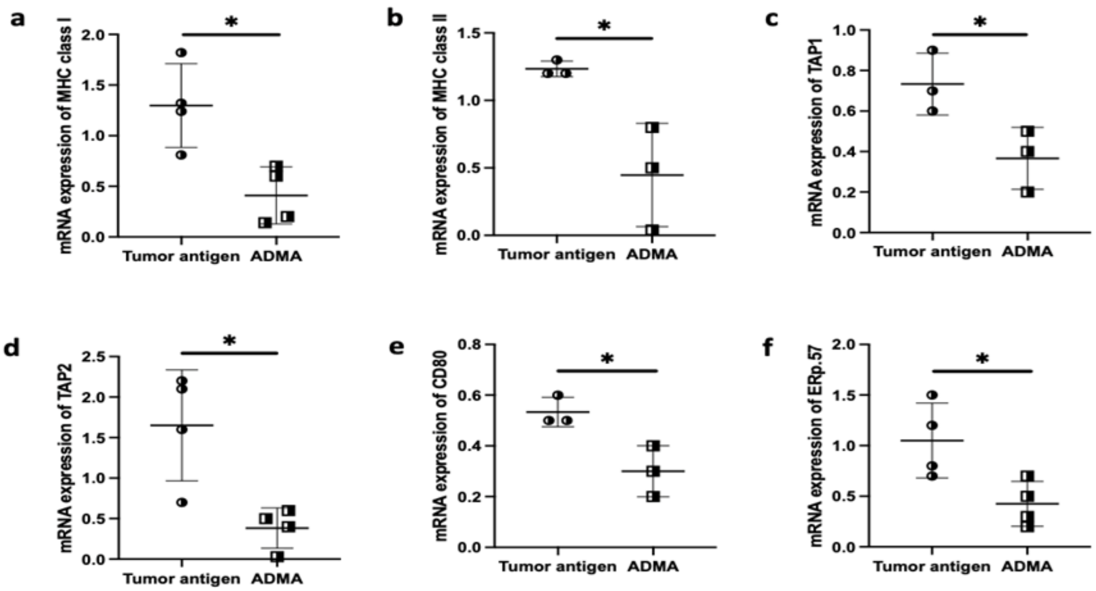

Supplementary Figure S2. Gene Expression Associated with Antigen Processing and Presentation in DC2.4 Cells Were Downregulated by ADMA. DC2.4 cells were treated with tumor antigens derived from mouse breast cancer Py230 cells in the presence or absence of ADMA for six days, followed by RNA isolation and RT-qPCR analysis. The expression of six key genes involved in antigen processing and presentation, including MHC I (A), MHC II (B), TAP1 (C), TAP2 (D), ERp57 (E), and CD80 (F) was assessed. Data represent pooled results from at least three independent experiments and are presented as mean ± SD. (*, $p < 0.05$; **, $p < 0.01$).

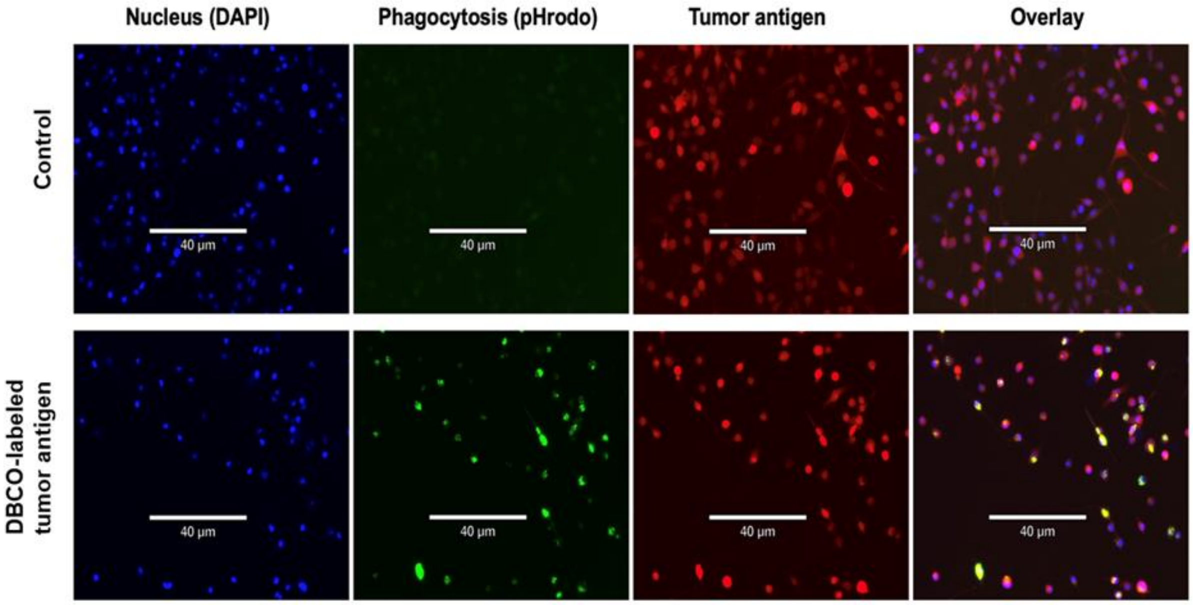

Supplementary Figure S3. DBCO conjugation does not affect the uptake of tumor antigens by DC2.4 cells. Triple immunofluorescence staining was used to visualize the subcellular localization of tumor antigens. Blue, green, and red signals label or indicate the nucleus, phagolysosomes, and tumor antigens, respectively (native tumor antigens shown in the top panel; DBCO-conjugated tumor antigens in the bottom panel). The DBCO-labeled antigens are efficiently internalized by DC2.4 cells and remain localized within the phagolysosomes.

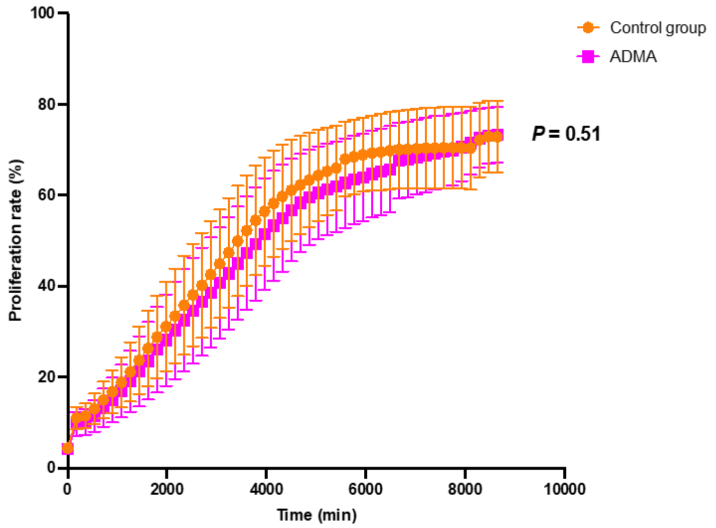

Supplementary Figure S4. The effects of ADMA on proliferation of DC2.4. ADMA was administrated to DC2.4 cells for 7 days and no significant impacts on the proliferation of DCs were observed in contrast to negative control group (N=7). Data are presented as mean ± SD.

Table S1. Primers used in this study.

Gene	Primer sequence (5' to 3')
MHC I-F	AACCCTCACCTTCATTTCCTGT
MHC I-R	TCAGACCCTGCCCTTTCTTAC
MHC II-F	CTGAGATGGGGTAAGGAGAGTG
MHC II-R	ACCAGAACAGCAACGGTCG
CD80-F	ATTGGTAGCAGAAGTGGGGTGT
CD80-R	CACAGACAGAGACTTTCCCCAC
ERp.57-F	GCAGAAATAGGCAGGGTGGT
ERp.57-R	TTCACGACTGACTTCCACGG
TAP1-F	CAGAGGTAGGTGAGACTGGGA
TAP1-R	GGTGAGAGCCAGAACCTTAC
TAP2-F	CCGGAGAATGTCTGCACTTTG
TAP2-R	ACCCCACCATCACCATTCTTC
